# Prediction of Optimal Reversal Dose of Sugammadex after Rocuronium Administration in Adult Surgical Patients

**DOI:** 10.1155/2014/848051

**Published:** 2014-02-11

**Authors:** Shigeaki Otomo, Hajime Iwasaki, Kenichi Takahoko, Yoshiko Onodera, Tomoki Sasakawa, Takayuki Kunisawa, Hiroshi Iwasaki

**Affiliations:** Department of Anesthesiology and Critical Care, Asahikawa Medical University, 2-1-1-1 Midorigaoka-higashi, Asahikawa, Hokkaido 078-8510, Japan

## Abstract

The objective of this study was to determine the point after sugammadex administration at which sufficient or insufficient dose could be determined, using first twitch height of train-of-four (T1 height) or train-of-four ratio (TOFR) as indicators. Groups A and B received 1 mg/kg and 0.5 mg/kg of sugammadex, respectively, as a first dose when the second twitch reappeared in train-of-four stimulation, and Groups C and D received 1 mg/kg and 0.5 mg/kg of sugammadex, respectively, as the first dose at posttetanic counts 1–3. Five minutes after the first dose, an additional 1 mg/kg of sugammadex was administered and changes in T1 height and TOFR were observed. Patients were divided into a recovered group and a partly recovered group, based on percentage changes in T1 height after additional dosing. T1 height and TOFR during the 5 min after first dose were then compared. In the recovered group, TOFR exceeded 90% in all patients at 3 min after sugammadex administration. In the partly recovered group, none of the patients had a TOFR above 90% at 3 min after sugammadex administration. An additional dose of sugammadex can be considered unnecessary if the train-of-four ratio is ≥90% at 3 min after sugammadex administration. This trial is registered with UMIN000007245.

## 1. Introduction

The recommended dose of sugammadex is determined based on the body weight of the patient and is set at 2 mg/kg of patient body weight for reversal of moderate neuromuscular block (NMB), that is, upon reappearance of the second twitch (T2) in train-of-four (TOF) stimulation, and at 4 mg/kg of patient body weight for reversal of deep NMB, that is, posttetanic count (PTC) 1-2. These recommendations have been defined by clinical consensus as the dose capable of exerting a reversal effect on NMB based on patient body weight, irrespective of individual differences. As a result, some patients administered the recommended dose receive more sugammadex than necessary. For example, moderate NMB is sufficiently reversed by 1 mg/kg sugammadex in some patients [1], and the recommended dose of 2 mg/kg sugammadex would constitute an overdose in such patients. An insufficient dose can lead to a return to NMB after temporary block reversal [2, 3], and avoiding underdosing is therefore essential. However, if it were possible to ascertain shortly after sugammadex administration that a patient required less than the recommended dose, adjustments could be made. Safe reduction of the sugammadex dose in individual patients would also contribute to reducing drug costs. In addition, if NMB is required again for reoperation after sugammadex administration, a dose of the blocking agent rocuronium or vecuronium sufficient to overcome the reversal effect of sugammadex must be administered to reinstigate blocking. In such situations, the amount of blocking agent required to reinstigate blocking could potentially be minimized if the appropriate sugammadex dose has been used. Recent research suggests that sugammadex, 1.0 mg/kg, rapidly and effectively reverses rocuronium-induced neuromuscular block which has recovered spontaneously to four twitches of TOF stimulation [4]. However, there is little research that considers the possibilities of safe reduction of the sugammadex dose for reversal of moderate or deep NMB. Against this background, we investigated the possibility of predicting whether the administered dose of sugammadex would be sufficient or insufficient by monitoring first twitch height of TOF (T1 height) and TOF ratio (TOFR) during recovery in adult patients receiving sugammadex. The primary objective of this study was thus to determine the point shortly after sugammadex administration at which sufficient or insufficient dose could be determined, using T1 height or TOFR as indicators.

## 2. Materials and Methods

The study was conducted at Asahikawa Medical University Hospital, Japan. The study protocol was approved by the ethics committee at the university and informed consent was obtained from all patients prior to enrolment. The protocol was reported to the UMIN clinical trials registry and given the code number UMIN000007245.

Subjects comprised 32 patients (18 male, 14 female) scheduled to undergo general anesthesia with tracheal intubation. ASA physical status was I-II, and patients with diabetes and neuromuscular disorders were excluded. Patients ranged in age from 20 to 82 years. Anesthesia was induced without premedication, using 1.5–2 mg/kg propofol, 0.3 *μ*g/kg/min remifentanil, and oxygen, and was maintained with 1.5–2% sevoflurane, 0.1–0.25 *μ*g/kg/min remifentanil, oxygen, and air. Rocuronium was administered at 0.6 mg/kg before tracheal intubation, then subsequently at 0.2-0.3 mg/kg when T2 reappeared in TOF stimulation. Routine monitoring included electrocardiography, pulse oximetry, and noninvasive blood pressure monitoring. Skin temperature of the hand was measured and kept above 33°C. Central temperature was monitored at the esophagus and kept above 35°C. During maintenance of anesthesia, end-tidal Pco2 was kept between 33 and 42 mm Hg. NMB was monitored using a TOF-Watch SX (Organon Ireland, Dublin, Ireland) and the accelography of the adductor pollicis muscle response to ulnar nerve stimulation was recorded. The TOF mode of stimulation (2 Hz; 0.2 ms) was applied at 15 s intervals throughout the procedure. Stabilization was achieved with a 5 s, 50 Hz tetanic stimulation. One minute after this tetanic stimulation, the fingers were fixated and completely immobilized, except for the thumb by using hand adapter (TOF-Watch Hand Adapter, Organon). After repetitive TOF stimulation for at least 3 min, calibration occurred by pressing the CAL button. Supramaximal stimuli were applied after automatic calibration of the device after an initial tetanic stimulus (CAL 2 mode). After calibration, the TOF-Watch SX was switched to the repetitive TOF stimulation again, which lasted until the end of anesthesia. NMB agent was not to be administered until at least 3 min after calibration, to check for correct setup. No preload was used, but we fixed the patient's arm and other 4 fingers throughout the entire duration of the procedure to avoid artifacts caused by movement. All neuromuscular monitoring data were transferred to a personal computer using a fiber-optic cable (TOF-Link), and saved using TOF-Watch SX Monitor software.

In the present study, a lower dose of sugammadex than what is recommended was administered as the first dose.

**Figure 1:**
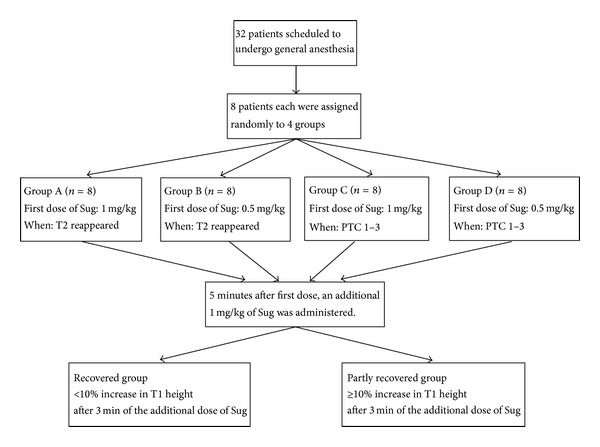
Patient flow through the study. Groups A and B received 1 mg/kg and 0.5 mg/kg of sugammadex (Sug), respectively, as a first dose when the second twitch (T2) reappeared in train-of-four stimulation (TOF), and Groups C and D received 1 mg/kg and 0.5 mg/kg of Sug, respectively, as the first dose at posttetanic counts 1–3 (PTC 1–3). Five minutes after the first dose, an additional 1 mg/kg of Sug was administered and changes in first twitch height of TOF (T1 height) and train-of-four ratio (TOFR) were observed. Patients were divided into a recovered group and a partly recovered group, based on percentage changes in T1 height after additional dosing. T1 height and TOFR during the 5 min after first dose were then compared.

Eight patients each were assigned randomly to 4 groups differentiated by extent of NMB remaining before first dose of sugammadex and size of first sugammadex dose. Groups A and B received 1 mg/kg and 0.5 mg/kg of sugammadex, respectively, as the first dose when T2 reappeared in TOF stimulation, while Groups C and D received 1 mg/kg and 0.5 mg/kg of sugammadex, respectively, as the first dose at PTC 1–3 in posttetanic stimulation. In each patient, T1 height and TOFR values were recorded every 15 s for 5 min after first dose of sugammadex. Five minutes after first dose, an additional 1 mg/kg of sugammadex was administered and changes in T1 height and TOFR were observed. Patients were divided into a recovered group and a partly recovered group based on percentage change in T1 height after the additional dose. T1 height and TOFR values every minute for 5 min after the first dose of sugammadex were then compared. The recovered group was defined as having <10% increase in T1 height after 3 min of the additional dose of sugammadex. The partly recovered group was defined as having ≥10% increase in T1 height after 3 min of the additional dose of sugammadex (Figure 1). After observations, all patients received a last additional dose of sugammadex so that the total dose matched the recommended dose stated in the product documentation, and recovery from NMB was confirmed in all patients after 30 min of the last additional dose. After recording all data, administration of sevoflurane and remifentanil was stopped and sufficient spontaneous breathing and patient response to verbal commands was confirmed. The airway tube was then removed.

The sample size was determined based on data for eight patients (two patients each for Groups A, B, C, and D) obtained from a preliminary study. There were 2 patients in the recovered group and 6 patients in the partly recovered group. T1 height at 3 min after first dose of sugammadex was 69.5 ± 10.6 in the recovered group and 40.0 ± 30.7 in the partly recovered group. Obtaining statistically significant results with *α* = 0.05 and a power of 0.8 required 28 patients in total. Considering dropouts, we enrolled 32 patients in total.

T1 height and TOFR were each compared between the recovered and partly recovered groups using an unpaired *t*-test. Characteristics of patients in Groups A to D were tested using a Kruskal-Wallis test for male-female ratio and one-way analysis of variance for age, height, weight, and body mass index. A significance level of *P* < 0.05 was set for each test.

## 3. Results

All enrolled patients completed the study. The breakdown of Groups A to D is shown in Table 1. No significant differences were seen between the characteristics of patients in Groups A to D (Table 1).

The recovered group included 6 of the 8 patients in Group A. The remaining 2 patients in Group A and all patients of Groups B–D were classed as partly recovered group (Table 2). T1 height and TOFR values from 1 to 5 min after the first dose and from 1 to 3 min after the additional dose of sugammadex in the recovered and partly recovered groups are shown in Tables 3 and 4. T1 height and TOFR values differed significantly between the recovered and partly recovered groups at all time points from 1 to 5 min after the first dose and from 1 to 3 min after the additional dose of sugammadex. In the recovered group, TOFR exceeded 90% in all patients at 3 min after the first dose of sugammadex administration. In the partly recovered group, none of the patients had a TOFR above 90% at 3 min after the first dose of sugammadex administration.

## 4. Discussion

Recovered group patients only came from Group A, and all patients of Groups B–D were in the partly recovered group. Six of the 8 patients in Group A were in the recovered group and 2 were in the partly recovered group, a finding in line with other studies on the optimum dose of sugammadex [2, 5]. Although some patients given 1 mg/kg sugammadex while in moderate NMB (when T2 reappeared in TOF) recovered to the same extent as those given 2 mg/kg, a large variation existed between patients and some only showed partial recovery.

Also this result was consistent with the findings of Duvaldestin et al. that sugammadex, 0.5 mg/kg and 1.0 mg/kg, could not provide rapid reversal of deep rocuronium-induced NMB under sevoflurane maintenance anesthesia [6]. T1 height and TOFR both differed significantly between the recovered and partly recovered groups at all time points from 1 to 5 min after the first dose of sugammadex administration. TOFR ≥ 90% is used as a clinical indicator of recovery from NMB after NMB reversal [7]. TOFR exceeded 90% in all patients in the recovered group at 3 min after the first dose of sugammadex administration. In the partly recovered group, no patients showed TOFR above 90% at 3 min after the first dose of sugammadex administration. We therefore concluded that an additional dose of sugammadex can be considered unnecessary if TOFR is ≥90% at 3 min after sugammadex administration.

We used TOFR as the optimum indicator of recovery from NMB after sugammadex administration for the following two reasons. When evaluating recovery from NMB after sugammadex administration with reference to T1 height, comparisons must be made with control values taken before administration of the neuromuscular blocking agent. This control value is taken before surgery and is thus susceptible to the influence of slight positional changes and so forth during surgery. We therefore considered that T1 height would be much more susceptible to measurement errors than TOFR. Also, TOFR is known to recover more quickly than T1 height with sugammadex-induced NMB reversal [8, 9], unlike in neostigmine-induced NMB reversal or spontaneous recovery. We therefore considered TOFR as a more suitable indicator for quickly determining the need for additional administration of sugammadex after the first dose.

Recurarization after insufficient doses of sugammadex has been reported in adults at around 20 min after sugammadex administration [3]. When postoperative NMB reversal is insufficient, the risk of postoperative respiratory complications such as aspiration is increased, potentially compromising airway maintenance [10]. The ability to determine the necessity for additional sugammadex doses at 3 min after first dose would largely remove the dangers of recurarization and contribute to cutting the risks of postoperative respiratory complications. The ability to determine at an early stage the suitability of additional sugammadex doses and NMB recovery could also lead to shorter times in the operating theatre and reductions in overall costs.

Sugammadex is a highly safe drug with few serious side effects. In phase I clinical trials, human subjects receiving a high intravenous dose of 96 mg/kg only showed taste disturbance as a characteristic adverse reaction [11]. Overdose in itself is thus not particularly problematic, and the risks of underdose would not seem to warrant a deliberate reduction in dose. However, if the sugammadex dose can be appropriately reduced, it may contribute to reductions in drug costs. For example, the recommended dose of sugammadex for reversal of moderate NMB in a patient weighing 120 kg is 240 mg (2 mg/kg). In this case, it would be necessary to use either two vials of 200 mg preparation or one vial of 500 mg preparation. If, however, it could be determined in a short period of time that a dose of 200 mg (1.67 mg/kg) was sufficient for the patient, only one 200 mg vial would be needed, thus reducing the drug cost.

Suitable reductions in sugammadex dose would also likely prove advantageous in the following situations.

Where reoperation requiring tracheal intubation and NMB is performed shortly after sugammadex administration, the benzylisoquinoline-based agents mivacurium and cisatracurium are suitable alternatives in some countries, as they do not bind to sugammadex. However, these agents are unapproved and therefore unavailable in other countries, including Japan. If suxamethonium is used, tracheal intubation can be performed rapidly, but subsequent maintenance of NMB is difficult. In these situations, the only alternative is to readminister rocuronium while remaining mindful of the fact that NMB reversal has been induced by sugammadex. At these times, keeping the sugammadex dose as low as possible becomes advantageous. According to Cammu et al. [12], when administering 0.6 mg/kg rocuronium, followed by the recommended dose of 4 mg/kg sugammadex during deep NMB of PTC 1-2, and then readministering rocuronium at 1.2 mg/kg at 5 min after sugammadex, the time for rocuronium to exert its action was 3.06 min (range, 1.92–4.72 min), notably slower than the original time for rocuronium to take effect when administered at 1.2 mg/kg. As sugammadex and rocuronium molecules normally bind in a 1 : 1 ratio, a lower amount of sugammadex in the body would be more conducive to reoccurrence of NMB when rocuronium is readministered.

The present study targeted patients ≥20 years old, and we do not know if similar results would be seen in children. Results might also differ in patients susceptible to peripheral nerve damage, such as diabetic patients. Sugammadex dose reduction in these patients is a topic for further research.

## 5. Conclusion

In adult patients with ASA physical status I-II, an additional dose of sugammadex appears unnecessary if TOFR is ≥90% at 3 min after initial administration. This suggests that safe and suitable reductions in sugammadex dose may be possible.

## Figures and Tables

**Table 1:** Patient characteristics.

	Sex (M/F)	Age (years)	Height (cm)	Weight (kg)	BMI (kg/cm^2^)
Group A (*n* = 8)	5/3	49.5 ± 18.9	167.0 ± 10.9	62.5 ± 8.6	22.4 ± 1.3
Group B (*n* = 8)	5/3	66.1 ± 9.4	160.4 ± 10.2	58.9 ± 9.6	23.1 ± 4.2
Group C (*n* = 8)	3/5	60.2 ± 19.7	158.3 ± 8.7	60.1 ± 13.3	24.0 ± 5.0
Group D (*n* = 8)	5/3	52.5 ± 23.0	163.0 ± 12.2	66.4 ± 12.5	25.0 ± 4.0

Values are given as mean ± SD or number. No significant differences are seen between groups.

Sug: sugammadex; BMI: body mass index.

**Table 2:** Breakdown of recovered group and partly recovered group.

	Sug dose timing	Sug first dose (mg/kg)	Recovered group/partly recovered group
Group A (*n* = 8)	T2	1	6/2
Group B (*n* = 8)	T2	0.5	0/8
Group C (*n* = 8)	PTC1-3	1	0/8
Group D (*n* = 8)	PTC1-3	0.5	0/8

Sug: sugammadex.

**Table 3:** T1 height at 1–5 min after first dose and at 1–3 min after the additional dose of sugammadex.

	T1 height 1 min after first dose	T1 height 2 min after first dose	T1 height 3 min after first dose	T1 height 4 min after first dose	T1 height 5 min after first dose	T1 height 1 min after additional dose	T1 height 2 min after additional dose	T1 height 3 min after additional dose
Partly recovered group (*n* = 26)	11.00 (0–43)	22.12 (0–68)	32.27 (0–75)	41.08 (0–94)	46.54 (0–92)	60.04 (0–109)	77.27 (14–125)	84.73 (37–133)
Recovered group (*n* = 6)	49.17 (29–75)	77.33 (50–99)	86.17 (62–108)	94.17 (70–116)	99.0 (86–115)	100.5 (85–115)	102.3 (86–115)	103.7 (88–117)

*P* values	<0.0001	<0.0001	0.0001	0.0008	0.0018	0.0085	0.0272	0.0438

Values are given as mean (min–max). T1 height values differed significantly between the recovered and partly recovered groups at all time points (*P* < 0.05).

T1: height first twitch height of TOF.

**Table 4:** TOF ratio at 1–5 min after first dose and at 1–3 min after the additional dose of sugammadex.

	TOF ratio 1 min after first dose	TOF ratio 2 min after first dose	TOF ratio 3 min after first dose	TOF ratio 4 min after first dose	TOF ratio 5 min after first dose	TOF ratio 1 min after additional dose	TOF ratio 2 min after additional dose	TOF ratio 3 min after additional dose
Partly recovered group (*n* = 26)	4.154 (0–30)	17.73 (0–63)	30.58 (0–83)	38.31 (0–89)	43.62 (0–90)	59.08 (0–102)	78.58 (0–109)	89.04 (23–116)
Recovered group (*n* = 6)	50.17 (28–64)	84.83 (70–97)	98.67 (92–110)	103.2 (94–116)	103.3 (98–115)	104.5 (100–117)	105.8 (102–116)	105.2 (101–118)

*P* values	<0.0001	<0.0001	<0.0001	0.0001	0.0009	0.0049	0.0244	0.0479

Values are given as mean (min–max). TOF ratio values differed significantly between the recovered and partly recovered groups at all time points (*P* < 0.05).

TOF ratio: train-of-four ratio.
